# UTILIZING PHYSICAL THERAPY RESIDENTS' AS HEALTH PROMOTION EXPERTS TO IMPROVE QOL FOR OLDER VETERANS

**DOI:** 10.1093/geroni/igac059.1633

**Published:** 2022-12-20

**Authors:** Susan Patel

**Affiliations:** Cincinnati VA Medical Center, Cincinnati, Ohio, United States

## Abstract

Gerofit, an evidence-based supervised exercise and health promotion program for older Veterans, has demonstrated success with its best practice model through the improved health, physical function, and well-being of participants. In 2017, Cincinnati VA Medical Center (CVAMC) launched an innovative approach to address the staffing needs of this wellness program by utilizing a Physical Therapy (PT) resident model for Gerofit group supervision/functional testing. As licensed PTs, residents engage in year-long advanced training in geriatrics across a variety of settings. They encounter adults who have unhealthy behaviors (lack of physical activity, smoking, poor nutrition, inadequate sleep, and stress), and demonstrate the knowledge/skills to reduce risk factors, prevent/treat chronic disease, prescribe physical activity/exercise, perform interventions consistent with the biopsychosocial paradigm, design/deliver individualized exercise prescriptions, and lead telehealth group exercises classes for Veterans. This presentation will explore how PT residents are in an ideal position to promote health/wellness in the Gerofit Program.

